# Internet of Things: A Review of Surveys Based on Context Aware Intelligent Services

**DOI:** 10.3390/s16071069

**Published:** 2016-07-11

**Authors:** David Gil, Antonio Ferrández, Higinio Mora-Mora, Jesús Peral

**Affiliations:** 1 Department of Computing Technology and Data Processing, University of Alicante, Alicante 03690, Spain; hmora@ua.es; 2 Department of Software and Computing Systems, University of Alicante, Alicante 03690, Spain; antonio@dlsi.ua.es (A.F.); jperal@dlsi.ua.es (J.P.)

**Keywords:** internet of things, big data, ontology, semantics, data mining with big data, services for big data, social internet of things, cloud computing

## Abstract

The Internet of Things (IoT) has made it possible for devices around the world to acquire information and store it, in order to be able to use it at a later stage. However, this potential opportunity is often not exploited because of the excessively big interval between the data collection and the capability to process and analyse it. In this paper, we review the current IoT technologies, approaches and models in order to discover what challenges need to be met to make more sense of data. The main goal of this paper is to review the surveys related to IoT in order to provide well integrated and context aware intelligent services for IoT. Moreover, we present a state-of-the-art of IoT from the context aware perspective that allows the integration of IoT and social networks in the emerging Social Internet of Things (SIoT) term.

## 1. Introduction

There are no doubts about how rapidly technology has grown in the last decade. Nowadays, a wide variety of devices, including sensor-enabled smart devices, and all types of wearables, connect to the Internet and power newly connected applications and solutions. On the one hand, the cost of technology has sharply decreased, making it possible for everybody to engage in sensing data. On the other hand, in every area we need to access the Internet, which delivers an amount of real time information. Furthermore, some of the environments are just online, like social media, where all the information is in the Cloud. As a result, new words as well as new expressions have appeared such as Big Data [1,2], Cloud Computing [3] or Internet of Things (IoT) [4,5,6], among others.

With the recent advances in radio-frequency identification (RFID), low-cost wireless sensor devices, and Web technologies, the IoT approach has gained momentum in connecting everyday objects to the Internet and facilitating machine-to-human and machine-to-machine communication with the physical world.

According to the Gartner Research report on the IoT, billions of connected things are already in use in 2015 and that number will reach 25 billion in just a few short years [7]. In addition, we can appreciate how easily the data can be generated currently to create a huge amount of information (e.g., Smart Cities [8], Business Intelligence [9], IoT [10]), generating big data [11,12].

There are several definitions or visions of IoT from different perspectives. From the viewpoint of services provided by things, IoT means “a world where things can automatically communicate to computers and each other providing services to the benefit of the human kind” [13]. From the viewpoint of connectivity, IoT means “from anytime, anyplace connectivity for anyone, we will now have connectivity for anything” [14]. From the viewpoint of communication, IoT refers to “a world-wide network of interconnected objects uniquely addressable, based on standard communication protocols” [15]. Finally, from the viewpoint of networking, IoT is the Internet evolved “from a network of interconnected computers to a network of interconnected objects” [16].

**Figure 1:**
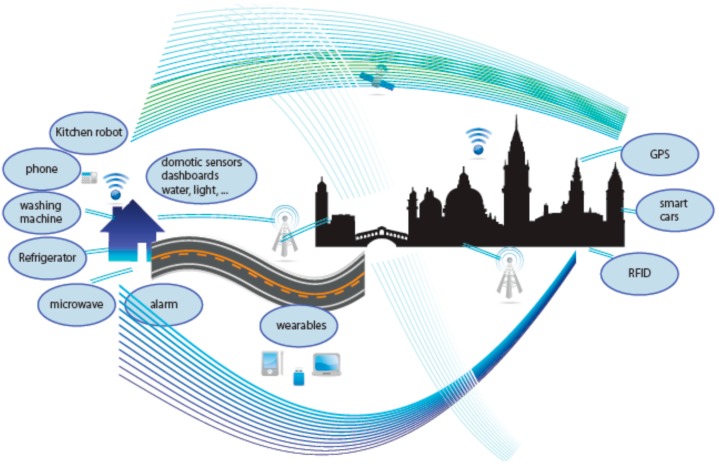
Illustration of data acquisition equipment in IoT.

As shown in Figure 1, data is processed differently in the IoT and traditional Internet environments (i.e., Internet of Computers). In the Internet of Computers, both main data producers and consumers are human beings. However, in the IoT, the main actors become things, which mean things are the majority of data producers and consumers. As pointed out by Ashton [10] IoT “has the potential to change the world, just as the Internet did”. Figure 1 shows the “things” able to generate data.

Although some reviews about IoT have been published recently (e.g., the most recent in [17]), they focus on high level general issues and are mostly fragmented. Therefore, the main objective of our paper is to carry out a complete survey of IoT, including the different types of technology for IoT; to review the previous surveys of IoT, including papers dealing with applications of IoT; and to indicate the number of services in order to incorporate context data to the information obtained both by sensors and/or created by the user (click worldwide) so as to enrich and give meaning to otherwise empty data.

The remainder of this paper is structured as follows: in the next section, the related work is reviewed. In particular, due to the number of surveys carried out, we establish classification criteria, which provide an index, facilitating easy access based on the key survey specifications. In the following section, we review the work on context aware computing in IoT applications. Thereafter, we explore the variety of services for the Internet of Things, including concepts such as data mining and big data in order to provide value and meaning to the simple data obtained by the IoT technology. Finally, we include a discussion with a summary of every section explaining our contribution to IoT.

## 2. Background on IoT Surveys

Given that some reviews of the IoT have been conducted recently, in this section the purpose is instead to perform a new survey, to summarize the most influential of reviews and work conducted recently in order to identify what are the shortcomings (if there are), the greatest opportunities and challenges that have not yet been addressed. In order to achieve this aim, we have grouped the IoT work in the following categories: general purpose IoT surveys, data oriented surveys, IoT vs. cloud computing surveys and IoT vs. data mining surveys. These categories are developed in the following subsections, which are concluded by a subsection that details potential IoT applications, and a final subsection that summarizes the open research issues for IoT.

### 2.1. General Purpose IoT Surveys

Diverse surveys give a picture of the current state of the art on the IoT. In general, they deal with issues of the basic features of IoT including architectures and technologies used. For example, Atzori et al. [6] provided the readers with a description of the different visions of the IoT paradigm coming from different scientific communities and reviews the enabling technologies and illustrates which are the major benefits of spread of this paradigm in everyday-life.

The work by [18] identifies the following key system-level features that IoT needs to support:

- Devices heterogeneity supported at both architectural and protocol levels.
- Scalability issues: (i) naming and addressing; (ii) data communication and networking; (iii) information and knowledge, and (iv) services provisioning and management.
- Ubiquitous data exchange through proximity wireless technologies.
- Energy-optimized solutions.
- Localization and tracking capabilities: to track the location (and the movement) of smart objects in the physical realm.
- Self-organization capabilities: making smart objects able to autonomously react to a wide range of different situations, in order to minimize human intervention.
- Semantic interoperability and data management among different applications: it is necessary to provide data with adequate and standardized formats, models and semantic description of their content (meta-data), using well-defined languages and formats.
- Embedded security and privacy-preserving mechanisms.

The survey in [19] reviews the three different phases with which the physical-cyber world interaction takes place: (i) collection phase (procedures for sensing the physical environment, collecting real-time physical data and reconstructing a general perception of it); (ii) transmission phase (mechanisms to deliver the collected data to applications and to different external servers); and (iii) process, management and utilization phase (by service-oriented architecture, cloud computing or peer-to-peer systems).

### 2.2. Surveys Oriented to Data

With regard to the characteristics of the data in IoT, surveys show the different types of data used and the main problems to be addressed related to them: generation, interoperability, storage, quality, and processing. The work in [20] presents five IoT technologies that are essential in the deployment of successful IoT-based products and services and discusses three IoT categories for enterprise applications used to enhance customer value. Because of the potential but uncertain benefits and high investment costs of the IoT, firms need to carefully assess every IoT-induced opportunity and challenge to ensure that their resources are spent judiciously. The authors of [21] study and discusses state-of-the-art techniques of IoT from the data-centric perspective.

A data stream, which is described in [21,22], is a sequence of data objects of which the number is potentially unbounded, continuously generated at a rapid rate. In the data stream, each data object can be described by a multi-dimensional attribute vector within a continuous, categorical, or mixed attribute space. In addition, there are some typical characteristics of data streams:

- Continuous arrival of data objects.
- Disordered arrival of data objects.
- Potentially unbounded size of a stream.
- Normally no persistence of data objects after being processed.
- Changing probability distributions of the unknown data generation process.

In general data stream processing one of the challenging issues is power constraints. In a typical sensor data processing system, techniques, including data aggregation, data compression, modeling and online querying, should be performed on-site or in-network to reduce communication cost [23].

RDF [24] as a general method for conceptual description and its predominant query language, graphs SPARQL [25,26], are standard in IoT and very often have been referenced in many studies.

Linked Data is a method for publishing structured data and interlink such data to make it more useful with the purpose of extracting RDF triples from unstructured data streams. Although the current Linked Open Data (LOD) cloud has tremendously grown over the last few years, it delivers mostly encyclopedic information (such as albums, places, and kings) and fails to provide up-to-date information [27]. Based on such observation, they develop RdfLiveNews, an approach that allows extracting RDF from unstructured (i.e., textual) data streams in a fashion similar to the live versions of the DBpedia and LinkedGeoData datasets.

In Table 1, we can see a data taxonomy representation, by identifying the intrinsic characteristics of IoT data and classify them into three categories, including data generation, data quality, and data interoperability. This three categories described in [21] are summarized in this Table 1.

In [21], the authors address storage issues in large scale systems. An interesting discussion arises about the solutions on how to replicate data across globally distributed data centres. For instance the idea of replicating all data to all locations requires using huge amounts of resources since users from different locations may have different data consumption needs [28]. In order to satisfy exceptional requirements of data storage in IoT, the distributed storage systems are crucial. There are three factors or requirements to be considered when designing a distributed storage system [1] which are consistency, availability and partition tolerance.

The work in [21] reviews the state-of-the-art research in IoT focusing on data, involving the processing of data stream, management and modeling of data storage. They identify the intrinsic characteristics of IoT data and classify them into three categories, including data generation, data quality, and data interoperability (see Table 1). They also discuss data stream research efforts that can help handle IoT data, including general data stream processing (analysing power constraints challenges), RFID data stream processing (analysing high-rate data streams processing challenges), and RDF triple stream processing (analysing linked data challenges). with regard to issues about data storage models, they examine traditional database management systems (DBMSs) and data warehouses, in contrast to large-scale storage in distributed environments, especially due to the arrival of the big data era. Moreover, they also discusses state-of-the-art techniques in searching things in the IoT environments, both in keywords based search on the textual description of the object whose sensor is attached, and in the collaborative search of things (e.g., through the collaboration of mobile phones).

### 2.3. Surveys about the Integration of Cloud Computing and IoT

Due to the relationships between IoT and cloud computing, some surveys show the connections between them and how they benefit each other. As the work in [29] states, cloud computing and IoT are often referenced as synonymous words. This is probably because they are complementary as they need each other, creating the Cloud-IoT concept. IoT can benefit from the virtually unlimited capabilities and resources of clouds to compensate its technological constraints (e.g., storage, processing and communication). On the other hand, clouds can benefit from IoT by extending their scope to deal with real world things in a more distributed and dynamic manner, and for delivering new services in a large number of real life scenarios.

The authors in [30] also consider that the Cloud will be a sort of intermediate layer to make connections between smart objects and applications. The goal is to make an efficient use of data and all the resources that these objects provide. They review the integration of IoT and cloud computing paradigms; provide an overview of the current state of research on this topic; and identify important gaps in the existing approaches.

Similarly, the work in [31] highlights how the IoT is capable to produce rapidly vast quantity of heterogenous data when there are millions of smarts obejcts providing data to cloud computing. The authors show a survey of integration components that consists of platforms and infrastructures for the Cloud and IoT middleware.

### 2.4. Surveys Oriented to Data Mining

The analysis of the large amount of data generated by IoT is a crucial problem. For this reason, new approaches of data mining for this kind of data are being developed. The survey presented in [32] summarizes the features of data mining for IoT. The authors present the relationship between data mining, Knowledge Discovery in Databases (KDD) and big data for IoT. The different mining technologies for IoT are also discussed in that work. The first reflexion is that the data from IoT are mostly too big and too tricky to be processed by the tools available currently [33,34,35,36]. Baraniuk in [37] describes that the bottleneck of data processing will be shifted from sensor to the data processing, communication, and storage capability of sensor. This observation also implies that the design and implementation issues of information system will be changed because of IoT.

Obviously, it is always much easier to create data than to analyze it. The flood of data it is certainly a serious problem of IoT. KDD systems available today and most traditional mining algorithms cannot be applied directly in order to process the large amount of data of IoT. Likewise, data mining technologies that exist currently work properly when they are applied to small scale IoT system. Therefore KDD and data mining technologies need to be redesigned for IoT in order to deal with large amount of data. New framework is presented in [32] to understand data mining algorithms [38,39,40,41,42].

The survey of data stream clustering in [22] presents an overview focused on data stream mining. The main goal is to find patterns and knowledge from huge amounts of unceasingly generated data. They review, with a deep analysis, data stream clustering algorithms to carry out unsupervised learning. They present a section of data stream clustering in practice presenting applications, data repositories and software packages.

### 2.5. Potential IoT Applications

**Figure 2:**
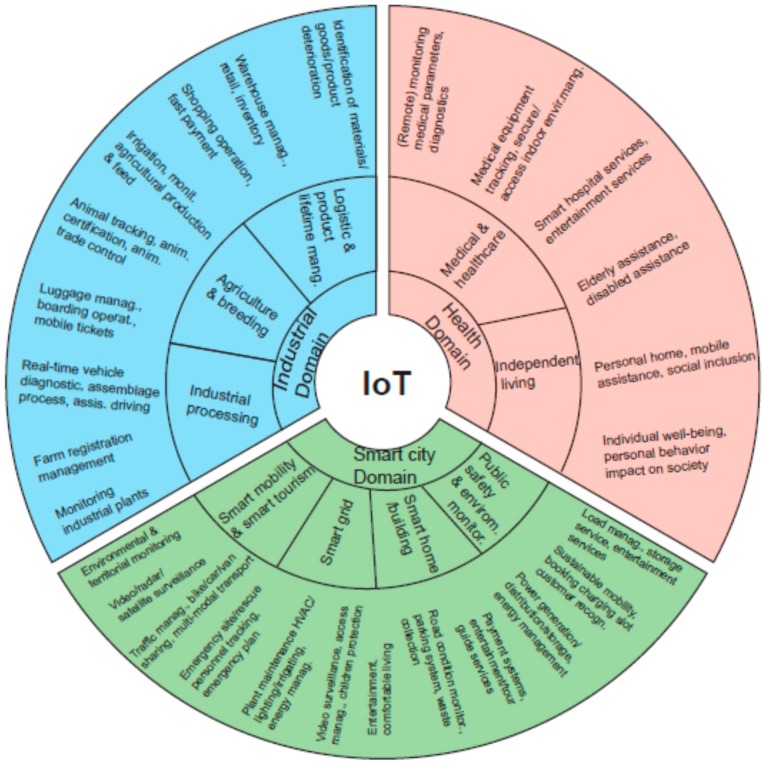
IoT application domains.

The huge potential of IoT is evident; therefore, many surveys have focused on IoT applications. From our point of view, we conclude that they could be divided into three big sets: smart city, industry and business, and health. The authors in [21] consider the following main groups of potential general IoT applications: smart cities and homes; environment monitoring; health; energy; business. In a similar way, the work in [6] groups these applications into the following domains: transportation and logistics domain; healthcare domain; smart environment (home, office, plant) domain; personal and social domain. In [43], the authors describe another IoT application: security and surveillance for enterprise buildings, shopping malls, factory floors, car parks and many other public places, as well as homeland security scenarios. The authors enumerate some specific applications such as the use of ambient sensors to monitor the presence of dangerous chemicals; sensors monitoring the behaviour of people may be used to assess the presence of people acting in a suspicious way; or personal identification by means of RFID or similar technologies. The work in [19] explores the IoT application domains and related applications is presented, where we can see a huge spectrum of application of IoT. Figure 2 shows the IoT application domains.

The main contribution of the survey presented in [44] is to show the state-of-the-art IoT in industries. In its background and current research of IoT section, RFID technology is considered as a foundational technology for IoT. RFID technology has been possible to identify, track and monitor any objects attached with RFID tags automatically [45]. RFID has been widely used in many and diverse areas, such as logistics, pharmaceutical production, retailing and supply chain management, since 1980s [46]. Another basic technology, contribute to the development of IoT, is the wireless sensor networks (WSN). WSN use interconnected intelligent sensors to sense and monitoring. Similarly to RFID, its applications are developed in many areas including environmental monitoring, healthcare, industrial monitoring, traffic monitoring, etc. [47,48].

Apart from RFID and WSN, many other technologies and devices are available today, such as barcodes, smart phones, social networks, and cloud computing. All these technologies are being used to establish an extensive network for supporting IoT [49,50,51,52].

Figure 2 illustrates some application scenarios. There exist some common challenges apart from the specific domains, which are: security, privacy, data integrity, analytics, mobility support, heterogeneity of objects and scalability. In addition to these challenges, there are technology specific challenges such as architecture, energy efficiency and quality of service. More applications can be found in [53] where a model-based methodology for the development of IoT applications in WSN is presented providing the development tools and software component libraries to describe the high-level application architecture, graphical definition, etc.

In [20], the authors categorize the IoT enterprise applications in three groups: (1) monitoring and control (where the smart home is known to be at the forefront of innovation regarding IoT monitoring and control systems); (2) big data and business analytics (to discover changes in customer behaviors and market conditions, to increase customer satisfaction, and to provide value-added services to customers); (3) information sharing and collaboration.

As mentioned, the survey reported in [44] focuses on the interest of using IoT technologies in various industries, as IoT is expected to offer promising solutions to transform the operation and role of many existing industrial systems such as transportation systems and manufacturing systems. Specifically, they review the IoT interests in agriculture, food processing industry, environmental monitoring, security surveillance, logistics, manufacturing, retailing, and pharmaceutics industries. They classify these interests in the following groups:

- IoT in the healthcare service industry: powered by IoT’s ubiquitous identification, sensing, and communication capacities, all objects in the healthcare systems (people, equipment, medicine, etc.) can be tracked and monitored constantly.
- Using IoT in food supply chain (FSC) to address the traceability, visibility, and controllability challenges. The so-called food-IoT comprises three parts: (a) the field devices such as WSN nodes, RFID readers/tags, user interface terminals, etc.; (b) the backbone system such as databases, servers, and many kinds of terminals connected by distributed computer networks, etc.; and (c) the communication infrastructures such as WLAN, cellular, satellite, power line, Ethernet, etc. As the IoT system offers ubiquitous networking capacity, all of these elements can be distributed throughout the entire FSC. Other work related to this issue are [54,55,56].
- IoT for safer mining production to sense mine disaster signals in order to make early warning, disaster forecasting, and safety improvement of underground production possible. For example, the effective communication between surface and underground in order to track the location of underground miners and analyze critical safety data collected from sensors to enhance safety measures. Another useful application is to use chemical and biological sensors for the early disease detection and diagnosis of underground miners, as they work in a hazardous environment.
- Using IoT in transportation and logistics to conduct real-time monitoring of the move of physical objects from an origin to a destination across the entire supply chain including manufacturing, shipping, distribution, and so on. Furthermore, IoT is expected to offer promising solutions to transform transportation systems and automobile services.
- IoT in firefighting to detect potential fire and provide early warning for possible fire disasters. By leveraging RFID tags, mobile RFID readers, intelligent video cameras, sensor networks, and wireless communication networks, the firefighting authority or related organizations could perform automatic diagnosis to realize real-time environmental monitoring.

In [57] they use IoT in the healthcare service industry. They insist on the importance of establishing an ecosystem in advance to take advantage optimally of opportunities. IoT provides new opportunities to improve healthcare [58]. Powered by IoT’s ubiquitous identification, sensing, and communication capacities, all objects in the healthcare systems (people, equipment, medicine, etc.) can be tracked and monitored constantly [59]. The healthcare-related information (logistics, diagnosis, therapy, recovery, medication, management, finance and even daily activity) can be collected, managed, and shared efficiently. The personal computing devices, such as laptop, mobile phone, tablet, wearables, etc. and mobile internet access (WiFi, optical fiber, 3G or 4G, LTE, etc.), the IoT-based healthcare services can be mobile and personalized [60]. They claim that since most societies are aging, IoT in the health sector could contribute to making elderly people’s lives easier.

There are other studies that focus in offering a framework for general applications. In the survey of Integration of cloud computing and Internet of Things [29], the authors explain that a cloud-based data access is able to cover the latency energy requirements of low power and the ubiquitous and fast access to data. In [61], the authors present the functional design and implementation of a complete WSN platform. The advantages of this proposal is not only the use for environmental monitoring IoT applications but also to take into account the requirements of design and specification such as low cost, high number of sensors, fast deployment, etc. The varied range of devices in IoT, with very heterogeneous capabilities whose response times are difficult to predict, are described in this work [62], which aims to respond to this issue by developing a computational model that formalizes the problem and that defines adjusting computing methods. The work presented in [62] describes the variety of devices in IoT. A computational model validate the problem in order to adjust computing methods.

In [63], López-Matencio proposes and studies a new node placement algorithm as this is a critical aspects of WSN design. The node placement determines sensing capacities, network connectivity, network lifetime and other capabilities of the WSN. WSNs are considered an enabling technology for unattended, long-lasting and rough terrain monitoring and have been widely studied in recent years [64] taking into account several considerations of design for optimization.

The authors of [65] present a computational architecture based on RFID Sensors. This study is applied to the traceability in smart cities. The aim of this distributed system is to obtain, represent and provide the flow and movement of people in densely populated geographical areas.

### 2.6. Open Research Issues for IoT

The general purpose surveys of IoT coincide in a set of open research issues for IoT, such as standardization, quality, security, or mobilization. For example, the survey by [6] enumerates the following: standardization, mobility support (e.g., scalability and adaptability to heterogeneous technologies), naming (i.e., mapping a reference to a description of a specific object), transport protocol, traffic characterization, authentication, data integrity, privacy and digital forgetting. The survey by [43], as a consequence of the previously described key system-level features that IoT needs to support, considers the following research challenges in IoT: computing, communication and identification technologies; distributed systems technology; distributed intelligence; security; data confidentiality; privacy; trust.

The work by [21] also discusses open research issues for IoT, but again focuses in data management issues. Specifically they enumerate the following issues: data quality and uncertainty (e.g., inconsistency detection); co-space data (e.g., to synchronize data in both real and virtual worlds); transaction handling (e.g., interaction between different networked computers/smart things with differing update policies); frequently updated timestamped structured (FUTS) data (e.g., real-time traffic reports, air pollution detection, temperature monitoring, and crops monitoring); distributed and mobile data (which makes IoT much more highly distributed and data intensive); semantic enrichment and semantic event processing (e.g., the progress of semantic Web to process and understand data through the semantic enrichment of sensing data); mining (e.g., the exploration and analysis of huge volume of IoT data); knowledge discovery (e.g., automatic extraction of relational facts from natural-language text and multi-modal contexts); security (e.g., RFID security); privacy (e.g., the lack of mechanisms to help people expose appropriate amounts of their identity information); social concerns (e.g., online social networks with personal things information may incur social concerns as well, such as disclosures of personal activities and hobbies).

Similarly, the survey reported in [44] focuses on research challenges for industrial use such as technology, standardization, security, and privacy. In particular, the authors consider as the main technical challenges: the design of a service-oriented architecture (SOA) for IoT (e.g., scalability issues, and situations in which service-based things might suffer from performance and cost limitations); to develop networking technologies and standards that can allow data gathered by a large number of devices to move efficiently within IoT networks; the lack of a commonly accepted service description language that facilitates the service development and integration of resources of physical objects into value-added services; the development of service discovery methods and object naming services; the integration of IoT with existing IT systems or legacy systems into a unified information infrastructure (e.g., the development of various middleware solutions in order to integrate IoT devices with external resources such as existing software systems and Web services); the development of green IoT technologies (e.g., energy-efficient techniques or approaches that can reduce the consumed power by sensors). Table 2 shows a summary of the different surveys studied along this section.

## 3. Context Aware IoT

Given the work reviewed in previous section about IoT surveys, in this section we explore the recent work developed on context aware intelligent services, in which the things in IoT dispose of enough intelligence to interact as social networks (as the capacities enumerated in [29]: self-configuration, self-optimization, self-protection, and self-healing). To the best of our knowledge, this is the first article that surveys the state-of-the-art of IoT from the context aware perspective that allows the integration of IoT and social networks in the emerging paradigm called Social IoT (SIoT) [68,69,70]. This section is concluded with the findings on this issue.

### 3.1. From Context Aware IoT to Social IoT

The context data add more meaning and value to the sensor data [71,72]. Borgia [19] highlights two IoT emerging research directions: IoT and social networks; and IoT and context-aware computing. The former reviews the interaction among smart objects following the social network paradigm. The main idea is that objects may have a social consciousness and may exhibit social behaviors allowing them to build their own social network of objects. This social network of objects can be exploited to enhance the trust level between objects that are “friends”, to guarantee a higher network navigability, and to make applications and services more efficient. Regarding the latter, the context awareness may provide a great support to process and store the big data, and to make their interpretation easier.

As the authors in [21] remark, they consider social concerns as a research challenge for IoT, specifically regarding the interaction of things in social networks. Likewise, Botta et al. [29] highlight the necessity of integrating social networking with IoT solutions, because they consider that there is a strong interest in using social networking to enhance communication among different IoT things. There is a trend for the move from IoT to a new vision named Web of Things that allows IoT objects to become active actors and peers on the Web.

The interpretation of SIoT in [73] attempts utilize users’ intuitive understanding of social networks to make the interconnected nature of IoT understandable and acceptable. In this line, the work in [74] makes use of an interactive IoT service on mobile devices. The concept of Social Web of Things (SWoT) is its foundation in order to do users capable of interacting with IoT in the same manner they use the social network services. As Rau et al. state, in SWoT, devices are presented as “beings” in social networks, with their interconnections compared to social relations. In addition, users can comment on the messages with natural language.

Atzori et al.’s work [75] identifies policies in order to establish as well as to manage social relationships to become a navigable social network. In addition, they describe an architecture for the IoT with the required functionalities of social network, and finally, they carry out simulations to study the characteristics of the SIoT network. The work in [76] introduces the idea of objects able to participate in conversations by discussing about the technology needed to guarantee an efficient interaction between the physical, social and virtual worlds. To ensure this interaction it is required the development of a data-centric architecture available for the people when and where they really require it.

Regarding the interconnections required between things, information retrieval (IR) techniques are introduced in the smart things. For example, Zhao et al. [77] propose an IR system based on topic discovery and semantic awareness in IoT environment. In this work and the one by [74], the state-of-the-art in IR for IoT is summarized. For example, the OCH system [78] allows users to query the current location of lost real-world objects; Dyser [79] is a search engine for the Web of Things, which allows real-world entities (i.e., people, places, and objects) to be searched by their current state; Snoogle [80] and Microsearch [81] are two systems that maintain an aggregate view of all sensors in a certain geographical area such as a room.

Context-aware approach is widely used for searching in IoT. For example, Covington et al. [82] proposed a role-based access control framework for context-aware applications. Giannikos and Kokoli [83] proposed and implemented a secure and context aware information lookup architecture for the IoT, which uses attributes to define access control policies, as well as, to semantically determine users and information items. In [84], the authors propose a context-aware service framework on top of IoT controlled systems, which is applied on the fault management process in electric power distribution networks. Their proposal takes automate actions depending on contextual information sensed from the IoT environment and received by the framework through its controlled systems. In [85], a context-aware and multi-service trust management system fitting the new requirements of the IoT is designed.

Information extraction (IE) is another artificial intelligence technique that is required for achieving the required high level of intelligence in things. For example in [77] proposal, the IE goal is to get metadata, such as the location and the topics, from the collected contents. These authors also use topic discovery tools to achieve topic self-adaptive retrieval, in which the theme information hidden in the texts is discovered and parsed out. Automatic classification and clustering for dividing the collected contents into two categories is also used in their approach: one category is the contents collected by topic focused collector (used to access data from predefined topic objects whose contents belong to a specific area) from topic websites or related IoT devices; the other category is the contents collected by general search engines-based collector (used to access general information on application-layer). Finally, natural language processing is an active research area used in this issue, which covers all the processing, understanding and interaction tasks [86,87,88,89].

### 3.2. Findings and Analysis on Context Aware IoT

After the reviewing of the work related on context aware IoT, next we summarize the main and open issues in this area:

1. There are two IoT emerging research directions: IoT and social networks; and IoT and context-aware computing.
2. The integration of IoT and social networks generates the emerging SIoT term.
3. There is a strong interest in using social networking to enhance communication among different IoT things.
4. There is a trend for the move from IoT to a new vision named Web of Things that allows IoT objects to become active actors and peers on the Web.
5. An agreed architecture is required, which includes the functionalities required to integrate things into a social network.
6. A further development of the technology is required to ensure an efficient interaction between the physical, social and virtual worlds by extending the Internet by means of interconnected objects.
7. Context-aware techniques are widely used for searching in IoT, such as information retrieval, information extraction and natural language processing techniques.

The main work reviewed in this section is summarized in Table 3.

## 4. Services for IoT

**Figure 3:**
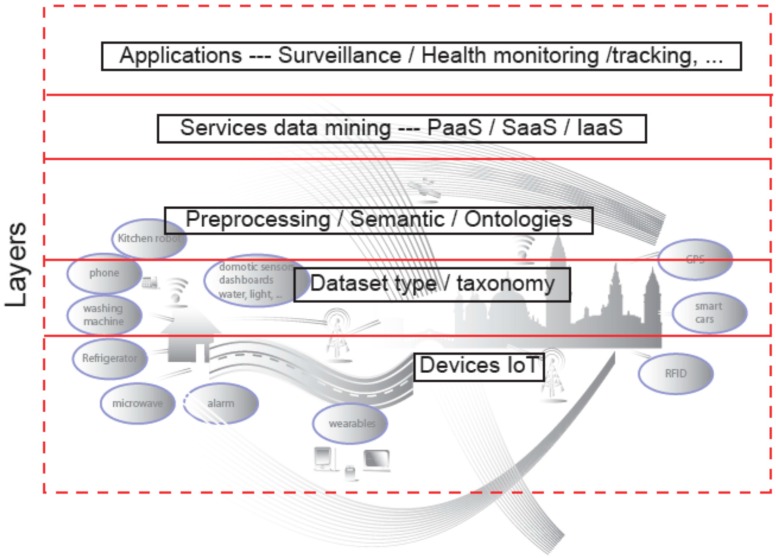
Overall architecture for IoT deployments and Applications.

Figure 3 shows the overall scheme and layers of the combination of IoT deployments, Cloud Computing technologies and end-user applications. As it can be seen, it is based on Figure 1 and it includes the different layers than can be integrated as the services we will explain in this section.

As it has been shown in previous sections, there are a lot of surveys related to the IoT technologies. However, the contribution of the review presented in this section is the global as well as comprehensive vision where all the techniques are integrated in order to provide services to the final users. In addition, many of those services will be able to be used by the applications developed in many diverse areas, such as surveillance, health, etc. Many of these applications were also reviewed in the former section. The layers of the Figure represent different acting areas of the services that will be explained in the next sections.

### 4.1. Service-Oriented Architecture (SOA) of IoT

The decentralized and heterogeneous nature of IoT (IoT connects different things over the networks) requires that the architecture provides IoT efficient event-driven capability. As a key technology in integrating heterogeneous systems or devices, SOA can be applied to support IoT because it is appropriate method to achieve integrity and reliability among all the heterogeneous sources provided by multitude of devices [6,43,90]. SOA has been fruitfully settled in research areas such as cloud computing, WSNs and vehicular network [90,91,92]. In [44] it is explained how the International Telecommunication Union recommends that IoT architecture consists of five different layers: sensing, accessing, networking, middleware, and application layers. It is very suitability to represent SOA architecture by means of layers. Other studies suggest other number of layers. Jia et al. [45] and Domingo [58] propose to divide the IoT system architecture into three layers instead the former five layers. These are perception layer, network layer, and service layer (or application layer). Atzori et al. [6] established a three-layered architectural model for IoT which consists of the application layer, the network layer, and the sensing layer. Liu et al. [93] designed an IoT application foundation that comprises a four-layered SOA of IoT, physical layer, transport layer, middleware layer and applications layer.

### 4.2. Service Models for Big Data Generated by IoT

The data produced by interconnected devices which sensing and interacting capabilities with the environment can be analyzed through data strategies. Its possibilities and opportunities are now endless. Today, the significance of IoT is not only a lot of devices interconnected. It is that the data generated from these devices is analysed upon new big data techniques to provide new perspectives on the environment around us. These scenarios of big data analysis on IoT systems provide enriched information to understand the dynamics of IoT applications, assist decision-making and management.

However, some of the operating characteristics of the IoT applications represent a challenge for IoT analysis: in first place, the communication needs of the connected things; secondly, the scale deployment of IoT applications can be very large; and finally, the variety of information sources and large amount of data generated in many of its implementation scenarios (smart city, smart transport, retail and logistics, etc.). In these cases, the ability of performing real-time analytics are out of reach of many organizations with limited resources. Here is where service models for big data come into play. This idea consists on providing big data services over the Cloud [5]. Currently, more and more companies are exploring cloud computing opportunities, using resources only when and where needed, as a way to reduce the cost and complexity of their IT services [94].

The definition provided by Patidar et al. defines cloud computing as a type of computing in which massively scalable IT-related capabilities are provided “as a service” by using Internet technologies to multiple external customers [95]. In other words, cloud computing is an IT paradigm for offering internet services. The adoption of cloud computing services is characterized by the following features [96,97]: on demand service, QoS guaranteed offer, autonomous system and scalability.

In general terms, this technology paradigm can be deployed according three service models: software as a service (SaaS), platform as a service (PaaS), and infrastructure as a service (IaaS) [97,98]. Infrastructure as a service (IaaS) provides IT hardware to the organizations. These resources meet the end-user requirements in terms of memory, computing and storage; platform as a service (PaaS) offers a development platform where end-users can build their own applications in the Cloud. Normally, PaaS contracts are complemented with IaaS to create cloud solutions for companies; software as a service (SaaS) delivers software or applications across the Internet. The end-users do not need to install and run the application on their local computers.

### 4.3. Data as a Service

Data and information are the most valuable resources in today information society. There are new service models that have arisen to provide specific services on data to the end-users thought Internet. In these scenarios, the Cloud providers are responsible for protect and supply the data in an affordable manner. In this way, useful data can be supplied to users on demand.

The Cloud, combined with other key technologies, enables companies to radically change how they service consumers and run their business [99]. A first step towards adopting cloud services is to perform data storage through an IaaS model and ubiquitous data access with a SaaS model. This case is known as ‘data as a service’ (DaaS) and it means that the data from multiple sources in several formats can be accessible via Cloud services. Allowing the data to be stored in the Cloud and be accessed without geographical and scalability limitations will remove many bottlenecks in bringing data-oriented innovations. DaaS aims to overcome drawbacks on data storage and access from repositories.

There are two approaches for DaaS: (a) on one hand, the data are collected and stored into the cloud by a third party and are rented to the organizations to perform analysis, graphs, maps, etc. For example, the data produced by a network of IoT devices in a smart city environment is provided on the Cloud to organizations which analyze them to generate added-value services to the citizen and city administrators; (b) On the other hand, the organizations decouple their data from applications and store them on the Cloud to be accessible for whole processes, and even, offer it to other partners. For example, the data generated by an ERP about the habits of their customers can be sold to other companies for offering commercial proposals or targeting marketing campaigns.

Combinations of they both can exists according to the business models around data [100]. In addition, a new concept of ‘open data’ has emerged. This term defines new ways to share information, which involves more transparency and wider access to information [101]. In this way, typical DaaS providers support from generic data assets, such as Amazon Web Services [102] or Microsoft Azure Data Marketplace [103].

The new scenarios introduced by the IoT paradigm involve that the data could be generated by a huge amount of ‘things’ all the time (in a city environment for example: weather stations, traffic cameras, metering devices, smart urban furniture, street lights, etc.) [8].

This changed reality of distributed sensing devices and their working contexts raise new challenges on data processing that must be addressed [99,104,105]. This list is not exhaustive and singles out the issues related with IoT and meaning extraction: structuring, interoperability, portability, decoupling:

- Structuring and classification: allows extracting meaningful content out of it and perform to make queries about it. In IoT scenarios, the data from sensors and other devices can be in an unstructured form. It is needed to define structures and protocols to add semantics such as tags, contextual info and other additional information.
- Interoperability: improves the data utilization. Collecting data from multiple sources can cause compatibility problems. The data can be heterogeneous in content and fashion. The standardization plays a key role in transforming the data towards a common interface to enable portability and facilitate accessing to it and re-use of data assets across different applications. This normalization produces data that can be handled by different services, applications and users.
- Decoupling data from applications: facilitates transparency, privacy and effectivity, and the developing of business models around the collected data. The data must be anonymized and decoupled from the sensing technology and its owner. For example, RFID tags on clothes.
- Integration: enables the data assimilation from different sources. The data is combined into valuable information. This process can involve adapting data fields and/or dimensionality reduction. Large scale data sets can be created by acquiring data from many sources, for example, from a network of IoT devices deployed on a smart city.

Table 4 summarizes a list of recent work on these issues only from a data management point of view as a representative example of the intense research that is currently being done.

In addition, there is work that specifically deals with sensing and IoT data acquisition issues. Table 5 describes an illustrative list of recent proposals.

Other challenges related with pricing the data, security of the data, data governance, and management of the services are also important issues in order to provide a quality data as a service.

### 4.4. Service Models of Data Mining with Big Data

In this section, we examine the different techniques and methods to be able to carry out experiment with the taxonomy of the IoT dataset. There are several initiatives that explore this objective [122,123,124,125,126]. After the study of those approaches, the idea is to offer services of data mining with all the data generated by IoT.

Table 6 includes a selection of the most common and employed programs for data mining and machine learning. Some of the software of the table is more advanced in the big data technologies than others, but all of them have approaches in order to deal with it. However, as will be explained later, data mining for big data (generated basically by IoT) is an outstanding issue and a big challenge for all the current technologies which are investing a lot of resources to deal with it.

Although many devices are mainly capable of generating data, some of the latest studies indicate that most of the things of IoT are supposed to have intelligence, thus are called “smart objects” (SO) and are assumed capable of being identified, sensing events, interacting with others, and making decisions by themselves [43,133,134,135]. One of the questions which arises in this survey is how do we convert the data generated or captured by IoT into knowledge. This is where KDD and data mining technologies come into play to find out the information hidden in the data of IoT. For this task a lot of researches have been using or developing effective data mining technologies for the IoT. The results described in [34,136,137,138] show that data mining algorithms can be used to make IoT more intelligent, thus providing smarter services.

### 4.5. Findings and Analysis on Services for IoT

After we have reviewed the services offered by IoT, here we present the opportunities and challenges:

1. The data generated from the different devices are analysed with new big data techniques to make easier the decision-making process. Data mining (DM) and machine learning (ML) techniques can be used.
2. Real-time analytics is possible through big data services over the Cloud.
3. The data are generated by a huge amount of ‘things’ all the time (in a city environment for example: weather stations, traffic cameras, metering devices, smart urban furniture, street lights, etc.). Depending on their characteristics, different techniques will be used: (1) natural language processing techniques (for example information extraction or question answering) to extract relevant information from textual unstructured information; (2) DM and ML techniques to make predictions about collected data; (3) Visualization techniques (dashboards or Google Analytics) to view graphically the data in order to facilitate the decision-making process.
4. Data from multiple sources in several formats are accessible via cloud services (data as a service, DaaS). The data are collected and stored into the Cloud and can be used/rented by other organizations to perform analysis, graphs, maps, etc.
5. Integration of information. It is a big challenge. The major idea is to integrate structured and unstructured information from different sources. Moreover, general/domain ontologies and reasoning techniques to match equivalent concepts among all the collected information can be used.

## 5. Conclusions

There are a considerable number of reviews about IoT and so far most of them have been conducted focusing on high level general issues. Furthermore, these articles do not specifically cover techniques on data processing and management, which is fundamentally critical to fully embrace IoT. In this paper, we have presented a systematic review of the diverse surveys of IoT. The huge volume of surveys that appear in the literature makes it easy to obtain a general picture of the current state of the art on the IoT but it is more difficult to discover the more promising parts of IoT, which is the key to succeeding when making a decision on IoT.

We review the current IoT technologies, approaches and models in order to discover what opportunities and challenges need to be met to make more sense of data. The revision of the available surveys in order to provide well integrated and context aware intelligent services for IoT has been carried out. Moreover, we propose an exhaustive state-of-the-art of IoT from the context aware perspective that allows the integration of IoT and social networks in the emerging social internet of things (SIoT) term, in which the things in IoT dispose of enough intelligence to interact as social networks. To the best of our knowledge, this is the first article that surveys the state-of-the-art of IoT from the context aware perspective that includes SIoT.

Finally, the diverse variety of services for IoT has been described. An overview and particular description of each service is has been developed concluding with a summary of those services and their opportunities.

This new field offers a lot of research challenges, but the main goal of this line of research is to make sense of data in any IoT environment. It has been pointed out that it is always much easier to create data than to analyze them. With this in mind, new conceptual modelling, (provided by ontologies, semantic, etc.) as well as new paradigms of data mining techniques, will be crucial to provide value and meaning to initially empty data.

## Figures and Tables

**Table 1:** IoT Data Taxonomy.

IoT Data Taxonomy	
Data Generation	
Velocity	Generated at different rates
Scalability	Large scale expectation
Dynamics	Mobile location, change Environments, connections intermittent.
Heterogeneity	Things generate data in different formats
Data Interoperability	
Incompleteness	Determine best data sources to address the incompleteness
Semantics	Injecting semantics into data is an initial step in IoT
Data Quality	
Uncertainty	Comes from different sources
Redundancy	Multiple measures
Ambiguity	Interpreted in different ways due to different data needs
Inconsistency	It can occur due: to missing readings, multiple sensors

**Table 2:** Summary of surveys.

References	Description	Main Proposals
[6,18,19]	General purpose IoT surveys	General visions of IoT. Key features and the driver technologies of IoT. Phases and interaction with the physical-cyber world.
[10,20,22,23,24,25,26,27]	Surveys oriented to data	Technologies in IoT-based products. Techniques of IoT from the data perspective. Data stream and data stream processing. RDF and SPARQL as method for conceptual description and query language respectively in IoT. Extraction of RDF triples from unstructured data streams.
[29,30,31]	Surveys about the integration of Cloud computing and IoT	Cloud computing and IoT are different technologies, but are complementary. Cloud becomes an intermediate layer between smart objects and applications. Integration components: cloud platforms, cloud infrastructures and IoT middleware.
[22,32,33,34,35,36,37,38,39,40,41,42,66]	Surveys oriented to data mining	Relationships between data mining, KDD and big data for IoT. Processing of big data and sensor information. Data mining algorithms. Data stream clustering.
[6,19,20,21,43,44,45,46,47,48,49,50,51,52,53,54,55,56,57,58,59,60,61,62,63,64,65,67]	Potential IoT applications	General IoT applications: smart cities and homes, environment monitoring, health, energy, business. Classification according several domains: transportation and logistics, healthcare, smart environment (home, office, plant), personal and social. Security and surveillance. Huge spectrum of applications of IoT. IoT applications in industries. RFID technology, wireless sensor networks, barcodes, smart phones, social networks, and cloud computing. Food supply chain. Different devices (capabilities) in IoT. Architectures based on WSN and RFID.
[6,19,21,43,44]	Open research issues for IoT	Standardization, mobility support, naming, transport protocol, traffic characterization, authentication, data integrity, privacy and digital forgetting. Computing, communication and identification technologies, distributed systems technology, distributed intelligence, security, data confidentiality, privacy and trust. Data quality and uncertainty, co-space data, transaction handling, Frequently updated timestamped structured data, distributed and mobile data, semantic enrichment and semantic event processing, mining, knowledge discovery, security, privacy and social concerns. Challenges for industrial use: technology, standardization, security and privacy. IoT and social networks and IoT and context-aware computing.

**Table 3:** Recent work on Context Aware IoT.

Work	Discussion
Internet of things marries social media [73]	Utilize users׳ intuitive understanding of social networks to make the interconnected nature of IoT understandable and acceptable
Social web of things of Chinese users [74]	An interactive IoT service on mobile devices based upon the concept of SWoT
The social internet of things [75]	It identifies appropriate policies for the establishment and the management of social relationships between objects in such a way that the resulting social network is navigable. They describe an architecture for the IoT that includes the functionalities required to integrate things into a social network
Social-driven internet of connected objects [76]	It introduces the idea of objects able to participate in conversations, and discusses about the technology required to ensure an efficient interaction between the physical, social and virtual worlds.
Topic-centric and semantic-aware retrieval system [74,77]	An IR system based on topic discovery and semantic awareness in IoT environment
The OCH system [78]	It allows users to query the current location of lost real-world objects
Dyser [79]	It is a search engine for the Web of things, which allows real-world entities to be searched by their current state
Snoogle [80] and Microsearch [81]	Systems that maintain an aggregate view of all sensors in a certain geographical area such as a room
Covington et al. [82]	A role-based access control framework for context-aware applications
Secure and context-aware information lookup for the IoT [83]	A secure and context aware information lookup architecture for the IoT
A context-aware dispatcher for the IoT [84]	A context-aware service framework on top of IoT controlled systems, which is applied on the fault management process in electric power distribution networks
A context-aware and multi-service approach [85]	A context-aware and multi-service trust management system fitting the new requirements of the IoT
Topic-centric and semantic-aware retrieval system for IoT [77]	Information Extraction techniques are applied to get metadata, such as the location and the topics, from the collected contents
Natural Language Processing for IoT [86,87,88,89]	NLP techniques applied on the processing, understanding and interaction tasks

**Table 4:** Recent work on issues for offering quality DaaS.

Work	Discussion	Main Proposals
XCLOUDX [99]	Data structuring, management, data services	Cloud-assisted data model
DaaS [106]	Large data sets challenges; decoupling data location	DaaS approach for abstracting the data location; fully decouple the data
Potential of Data [101]	Open standards, interoperability	Best practices recommendations to enhance manageability, discovery, accessibility and usability
Open Data as Universal Service [101]	Open data, interoperability	New roles for data queries
Data services [107]	SOA architectures and Cloud computing for data processing	Conceptual framework for service oriented decision support systems
Data management in the Cloud [108]	Data management and data analysis in the Cloud	Parallel databases features for cloud data computing environments
Data as a Service Framework [109]	Integration, interoperability, data services	A framework for providing reusable enterprise data services
Demods [110]	Service and data discovery, data integration	Model for data-as-a-service
DaaS concerns [111]	Data services issues	Modelling concerns for DaaS. Evaluation of current Daas publishing
Data Integration [112]	Data services, interoperability, integration	Ontology-based framework for describing and integrating data
Privacy-Preserving DaaS [113]	Decoupling, privacy preserving, anonymization	A framework for privacy-preserving data-as-a-service
SOA data mashup [114]	Data services, privacy preserving, data integration	SOA architecture for high-dimensional private data mashup
Data integration [115]	Data integration, multidimensional data	Semantic foundation for multidimensional data integration, query operators and optimization

**Table 5:** Recent work on sensing and IoT data acquisition issues.

Work	Discussion	Main proposals
Sensor Data as a Service [116]	Sensor network and service platforms	Sensor data federation as a service featuring interoperability, reusability and decoupling
Sensor Data Services Query [117]	Data structuring, sensor data services	Service model for query sensor data
DaaS IoT [118]	Data structuring, integration, dimensionality reduction, data services	Data-as-a-service framework for IoT
Service model for smart cities [8]	Service model, data services, data acquisition, privacy preservation, decoupling	Model for sensing as a service supported by Internet of Things
CityWatch [119]	Sensor data, data acquisition, interoperability, integration	Data sensing and sensor dissemination framework
IoT Data distribution [120]	Data acquisition, interoperability, integration	Data framework to distribute context data
IoT Cloud Computing [29]	Integration, data Interoperability, structuring, large scale, interoperability	Analyssis of cloud IoT paradigm and identify the open issues and future directions in this field
Data Analysis as a Service [121]	Data acquisition, integration, interoperability	Infrastructure for storing and analyzing data from the Internet of Things

**Table 6:** Data Mining for IoT.

Work	Name	Description
[127,128]	R	Open source programming language and software environment, is designed for data mining/analysis and visualization. It is used for data exploration, statistical analysis, and drawing plots.
[40,123,129,130]	Weka	Weka is a free and open-source machine learning and datamining software written in Java. Weka provides such functions as data processing, feature selection, classification, regression, clustering, association rule, and visualization, etc.
[131]	Rapid-I Rapidminer	Rapidminer is an open source software used for data mining, machine learning, and predictive analysis. Data mining and machine learning programs provided by RapidMiner include Extract, Transform and Load (ETL), data pre-processing and visualization, modeling, evaluation, and deployment.
[132]	KNMINE	It is a user-friendly, intelligent, and open-source-rich data integration, data processing, data analysis, and data mining platform.
